# Analysis of Serotonin in Human Feces Using Solid Phase Extraction and Column-Switching LC-MS/MS

**DOI:** 10.5702/massspectrometry.A0081

**Published:** 2020-03-31

**Authors:** Yukiko Hirabayashi, Kiminori Nakamura, Tsuyoshi Sonehara, Daisuke Suzuki, Satoru Hanzawa, Yu Shimizu, Tomoyasu Aizawa, Koshi Nakamura, Akiko Tamakoshi, Tokiyoshi Ayabe

**Affiliations:** 1 Research and Development Group, Hitachi, Ltd., 1–280 Higashi-koigakubo, Kokubunji, Tokyo 185–8601, Japan; 2 Faculty of Advanced Life Science, Hokkaido University, Kita 21, Nishi 11, Kita-ku, Sapporo, Hokkaido 001–0021, Japan; 3 Graduate School of Life Science, Hokkaido University, Kita 21, Nishi 11, Kita-ku, Sapporo, Hokkaido 001–0021, Japan; 4 Faculty of Medicine, Hokkaido University, Kita 15, Nishi 7, Kita-ku, Sapporo, Hokkaido 060–8638, Japan; 5 Graduate School of Medicine, University of the Ryukyus, 207 Uehara, Nishihara, Okinawa 903–0215, Japan

**Keywords:** serotonin, feces, column-switching LC-MS/MS, solid phase extraction

## Abstract

Serotonin, an important neurotransmitter, is produced mainly in intestines, and serotonin levels in feces can be an indicator of the intestinal environment. Human feces, however, contain a large amount of contaminants, which vary widely owing to food contents and the intestinal environment, and these contaminants would be expected to interfere with the determination of serotonin levels in human feces. To remove these contaminants and determine serotonin levels, we developed a new method using solid phase extraction (SPE) and column-switching LC-MS/MS. Serotonin, labeled with a stable isotope, was added to human feces samples prior to SPE as an internal standard to correct for individual differences in matrix effects. The recovery rate for SPE was 55.9–81.0% (intraday) and 56.5–78.1% (interday) for feces from two subjects. We analyzed 220 fecal samples from 96 subjects including 76 pregnant and post-delivery women. The endogenous serotonin content per unit weight of dried feces was 0.09–14.13 ng/mg for pregnant and post-delivery women and 0.30–9.93 ng/mg for the remaining subjects.

## INTRODUCTION

In recent years, the gut has attracted attention as a “second brain.” Investigations of bacterial flora in the human gut using the 16S rRNA-based approach to examine gut microbiota in feces^1,2)^ have become popular in studies of relations between gut microbiota to diseases,^3)^ diet,^4)^ age and geography,^5)^ cognition in infants,^6)^ *etc*. Metabolomic analyses of fecal metabolites using nuclear magnetic resonance^7)^ and mass spectrometry^8,9)^ have been reported as well. These studies have mainly focused on the composition and function of gut microbiota and analyses of substances derived from the hosts in feces have also been reported. For example, a quantitative analytical method using a sandwich enzyme-linked immunosorbent assay for α-defensins, which are produced by Paneth cells and are thought to regulate the growth of gut microbiota, has been developed,^10)^ and studies concerning the involvement of α-defensins in diseases and health have been reported.^11,12)^

The “brain–gut interaction” in which the brain and gut affect each other is important because of its implications in various diseases.^13,14)^ Serotonin, which is produced in both the brain and the gut and functions in each organ, also plays a key role in brain–gut interactions.^14)^ In addition, the results of previous studies suggest that serotonin in the context of brain–gut interactions can participate in mood disorders, including depression. For example, patients with depression tend to have constipation^15)^ and constipation is related to serotonergic regulation as shown by patients with constipation-predominant irritable bowel syndrome, which is accompanied by an impaired release of serotonin.^16)^ Serotonin has also been implicated in depression, and it has been reported that blood serotonin levels are reduced in patients suffering from major depressive disorder with melancholia.^17)^ It has also been reported that blood serotonin levels are correlated with tension/anxiety in pregnant and post-delivery women who are at high risk for mood disorders^18)^ and are generally prone to constipation.^19)^

Serotonin is mainly produced by enterochromaffin cells in the gut, and about 90% of the total amount of serotonin in the body is found in the gut.^20)^ Therefore, monitoring the production of serotonin (serotonin levels) in the gut will be of interest in studies of physiological and mental conditions, and for potentially detecting early stages of depression and mood disorders. Serotonin produced by the gut may be detected in feces. Serotonin in feces presumably reflects the status of serotonin production in the gut. However, determining trace substances in feces is very difficult due to large amount of contaminants that are present. In addition, contaminants in human feces may vary widely depending on the food being ingested. There are a few reports on the analysis of serotonin in rat feces using high performance liquid chromatography (HPLC)^21)^ and capillary electrophoresis,^22)^ but, to our knowledge, the measurement of serotonin in human feces has not been reported to date.

In this study, we develop an analytical method for the quantification of serotonin in human feces using solid phase extraction (SPE) and column-switching liquid chromatography-tandem mass spectrometry (LC-MS/MS), and serotonin levels in human feces from adult subjects, including pregnant and post-delivery women, are reported.

## EXPERIMENTAL

### Chemicals and materials

Serotonin Creatinine Sulfate Monohydrate (serotonin) for the serotonin standard and isotope-labeled Serotonin Creatinine Sulfate Monohydrate (α, α, β, β-d_4_, 98%, serotonin-d_4_) for the internal standard (IS) were purchased from Tokyo Chemical Industry Co., Ltd. (Tokyo, Japan) and Otsuka Pharmaceutical Co., Ltd. (Tokyo, Japan), respectively. Each of these materials was dissolved in a water–methanol mixture (water/methanol=40/60%, v/v) to prepare a 1 mM stock solution, which was then diluted to prepare samples for method evaluation, quality controls, and calibration curves.

Dipotassium hydrogen phosphate and potassium dihydrogen phosphate were purchased from FUJIFILM Wako Pure Chemical Corporation (Osaka, Japan). Two aqueous solutions of the same concentration of dipotassium hydrogen phosphate and potassium dihydrogen phosphate were mixed at a ratio of 6 : 4 to prepare a 10 mM phosphate buffer at pH 6.8. Acetonitrile, formic acid, and 25% ammonia water were purchased from FUJIFILM Wako Pure Chemical Corporation. A 7.5% ammonia solution (water/methanol=30/70%, v/v) was prepared by mixing 25% ammonia water and methanol at a ratio of 3 : 7.

We used spin centrifuge columns for the SPE. The cation exchange column, MonoSpin© CBA, was purchased from GL Science Inc. (Tokyo, Japan).

### Fecal samples

Human fecal samples were obtained from two groups of subjects.

The first group (Group 1) consisted of 20 adult volunteers recruited from the staff and students at Hokkaido University (aged 21–60, 18 men and 2 women) with informed consent. The first set of samples were collected from 12 subjects, and the second set of samples were collected after 10 months from 16 subjects (samples from 8 subjects were collected both times), for a total of 28 samples. These were mainly used for method development and validation.

Fecal samples, collected in a dedicated container with a lid, were first stirred, dispensed, and frozen at −80°C, then lyophilized and powdered.^10)^ Most were lyophilized by a freeze dryer FDU-2110 (Tokyo Rikakikai Co., Ltd., Tokyo, Japan) and powdered using a crusher Multi Beads Shocker® MB 2000 (Yasui Kikai Corporation, Osaka, Japan), then stored at −80°C. A portion of each sample was lyophilized and powdered by TechnoSuruga Laboratory Co., Ltd. (Shizuoka, Japan), and used for the quantification of serotonin.

The second group (Group 2) consisted of 76 pregnant and post-delivery women (aged 22–40) living in Iwamizawa City, Hokkaido who agreed with the purpose of the study and had joined the “Maternal and Child Health Research Program” at Iwamizawa City, in collaboration with Hokkaido University.^23)^ A total of 192 fecal samples were obtained at either of four time points: 65 during pregnancy (mid pregnancy), 59 within one week after delivery, 41 at one month after delivery, and 27 at 8–9 months after delivery. Fecal samples collected in a dedicated container with a lid, lyophilized and powdered by TechnoSuruga Laboratory Co., Ltd.

The study was approved by the Ethics Committee of Hokkaido University and that of Hitachi, Ltd.

### Serotonin solution preparation

Three serotonin solutions were prepared: for use in preparing calibration curves, for quality control (QC), and for elution.

Since endogenous serotonin cannot be removed from feces, water was used as the substitution matrix for samples for the calibration curve and samples for QC. A stable isotope-labeled serotonin-d_4_ was used as the IS. First, 1-mM stock aqueous solutions of serotonin and serotonin-d_4_ were prepared. For calibration curves, the stock solutions of serotonin were diluted to 0, 0.005, 0.01, 0.05, 0.1, 0.5, 1.0, 2.0, 3.0, and 5.0 μM, and the stock solutions of serotonin-d_4_ were added to these solutions at a final concentration of 0.5 μM.

For QC, 0.01, 0.1, 0.5, and 1 μM serotonin solutions containing 0.5 μM serotonin-d_4_ were prepared using water as a substitution matrix following the same procedure.

The eluting solution added to the powdered feces consisted of a 10 mM phosphate buffer (pH 6.8), 20% acetonitrile and 0.5 μM serotonin-d_4_ as the IS.

### Fecal sample preparation and solid phase extraction

A 20 mg sample of powdered feces was collected in a microtube, and 400 μL of an eluting solution (10 mM phosphate buffer at pH 6.8, 20% acetonitrile) containing 0.5 μM serotonin-d_4_ was added to the powdered feces. After suspending the powdered feces in the eluting solution, the suspensions (hereafter, referred to as the fecal solution) were mixed overnight using a microtube mixer (MT-400, TOMY SEIKO Co., Ltd., Tokyo, Japan). The fecal solution was then centrifuged at 20,000 g and 4°C for 20 min using a centrifuge (MX series, TOMY SEIKO Co., Ltd., Tokyo, Japan), and a 200 μL aliquot of the supernatant was introduced into an SPE spin column (for strongly basic substances, MonoSpin® CBA, GL Sciences Inc.).

The SPE procedure was as follows. For equilibration, 200 μL of 10 mM phosphate buffer (pH 6.8) was introduced into the SPE spin column and the spin column was centrifuged at 10,000 g at 4°C for 1 min. A 200 μL aliquot of the supernatant of the fecal solution and 400 μL of the 10 mM phosphate buffer were mixed and introduced into the SPE spin column, which was then centrifuged. After washing the column with 200 μL of the 10 mM phosphate buffer, 300 μL of a 7.5% ammonia solution (water/methanol=30/70%, v/v) was introduced into the column for extraction and centrifugation. The extracted solution containing serotonin was collected in a sample tube and dried using a centrifugal evaporator (SPD1030, Thermo Fisher Scientific Inc., MA, USA). The dried sample was re-dissolved in 200 μL of water and analyzed by LC-MS/MS.

### LC-MS/MS analysis

Analysis was performed using an LC-MS/MS consisting of an ultra-high performance liquid chromatograph (LaChromUltra, Hitachi High-Tech Corporation, Tokyo, Japan) and a triple quadrupole mass spectrometer (3200 Q TRAP, SCIEX, MA, USA). The mass spectrometer was operated in the positive ion mode of electrospray ionization (ESI) at 5.5 kV and the multiple reaction monitoring (MRM) mode. Detailed conditions for the mass spectrometer for the MS/MS analysis are shown in Table 1.

**Table 1:** Table 1. Optimal conditions for the MS/MS analysis of serotonin and serotonin-d_4_.

Ionization	ESI (+)
Voltage of ionization	5.5 kV
ESI heater temperature	550°C
Nebulizer gas pressure	80 (a.u.)
Auxiliary gas pressure	70 (a.u.)
Curtain gas pressure	10 psi
Collision gas pressure	5 psi
Declustering potential	35 V
Entrance potential	5 V
Collision energy	15 V
Collision cell exit potential	2 V
MRM transition (Q1 → Q3):	
Serotonin	*m*/*z* 177.2→160.1
Serotonin-d_4_	*m*/*z* 181.2→164.1

**Figure 1:**
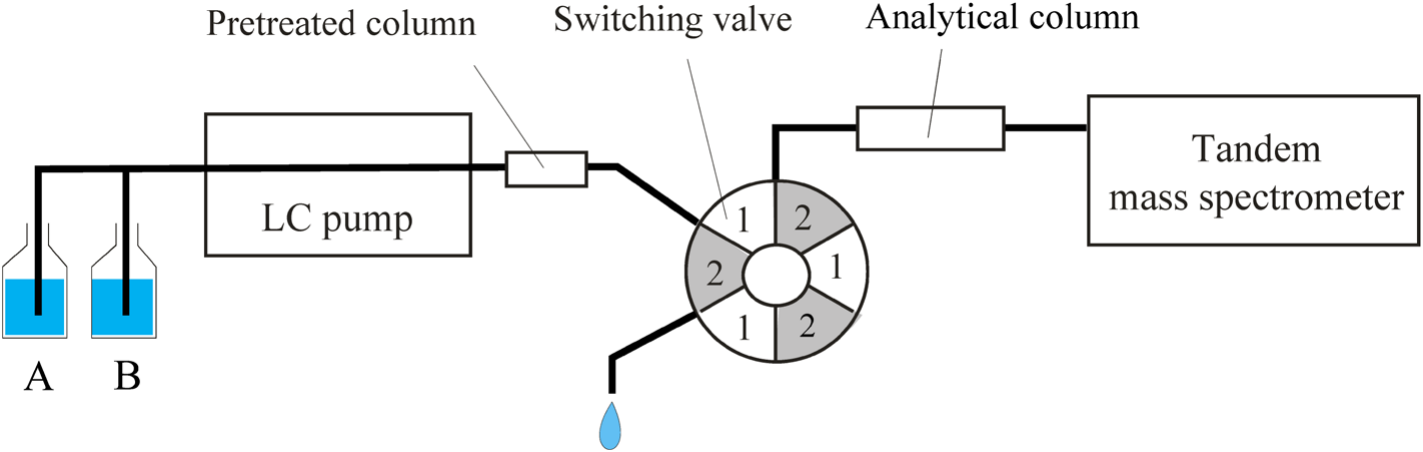
Fig. 1. Column-switching LC-MS/MS setup.

LC was performed using a C18 analytical column (2.1 mm I.D., 100 mm long, 3 μm particle diameter; J-Pak Wrapsil C18, JASCO Corporation, Tokyo, Japan) and a precolumn (4.0 mm I.D., 20 mm long, 5 μm particle diameter, CAPCELL PAK MF Ph-1, Osaka Soda Co., Ltd., Osaka, Japan) for protein removal. A switching valve (3011, Osaka Soda Co., Ltd.) was located between these columns, as shown in Fig. 1. The mobile phase was a mixture of two 0.1% formic acid acetonitrile solutions: solution A, 2% (v/v) acetonitrile, solution B, 100% acetonitrile. The flow rate was 200 μL/min and 10 μL of the sample solution was injected into the LC-MS/MS system. At the time of sample injection, the ratio of solution A and solution B was 82 : 18. The LC gradient program is shown in Table 2. Briefly, the valve was switched from position 1 to 2 at 0.8 min after the sample injection to drain proteins and contaminants, the valve was then returned to position 1 at 1.3 min, and the samples containing serotonin were delivered to the analytical column connected to the mass spectrometer.

**Table 2:** Table 2. Column-switching program.

Time (minutes)	Valve position	LC condition		
		A: 0.1% Formic acid (2% MeCN)	B: 0.1% Formic acid (100% MeCN)	Flow rate (μL/min)
0.0	1	82%	18%	200
0.8	2	82%	18%	200
1.3	1	82%	18%	200
3.9	1	82%	18%	200
4.0	1	10%	90%	200
4.3	1	10%	90%	200
4.4	1	82%	18%	200
10.0	1	82%	18%	200

Data acquisition, peak area calculations, and the preparation of calibration curves were conducted using Analyst© software (SCIEX).

### Recovery rate and matrix factor

The recovery rate was evaluated using 0.01, 0.1, 0.5 and 1 μM serotonin-d_4_. The fecal solution containing each concentration of serotonin-d_4_ (pre-added solution) was processed by SPE and analyzed by column-switching LC-MS/MS. The fecal solutions without serotonin-d_4_ (0 μM) were also processed by SPE and the serotonin-d_4_ was then added to the processed samples so that the serotonin-d_4_ concentration became 0.01, 0.1, 0.5 and 1 μM, respectively (post-added solutions). The post-added solutions were also analyzed by column-switching LC-MS/MS. The recovery rate (%) was calculated by comparing the serotonin-d_4_ peak area for the pre-added solutions with that for the post-added solutions.

The matrix factor (MF) (%) was calculated by comparing the serotonin-d_4_ peak area for 0.5 μM post-added solutions with that for 0.5 μM aqueous solutions of serotonin-d_4_.

## RESULTS AND DISCUSSION

### Method validation

The calibration curve constructed from the peak area ratio of serotonin against 0.5 μM IS serotonin-d_4_ showed good linearity between 0.005 and 5 μM of serotonin concentrations (*r*=0.999). The LLOQ was 0.005 μM. Table 3 shows the accuracy and precision for 0.01, 0.1, 0.5, and 1 μM QC samples using water as the substitution matrix.

**Table 3:** Table 3. Accuracy and precision of the column-switching LC-​MS/MS method.

Concentration of QC samples (μM)	Accuracy (%)		Precision, CV (%)	
	Intraday (*n*=6)	Interday (*n*=5)	Intraday (*n*=6)	Interday (*n*=5)
0.01	+3.4	+2.1	1.4	1.7
0.1	+1.3	+0.1	0.4	1.1
0.5	+3.0	+2.1	0.4	1.0
1	+2.6	+1.7	0.4	0.9

Matrix effects of the feces and the recovery rate of SPE were evaluated using stable isotope-labeled serotonin-d_4_. No interfering contaminant ions derived from feces with protonated serotonin-d_4_ were detected.

Two fecal samples from subjects 3 and 5 in the first collection series of Group 1 were selected to evaluate the recovery rate of SPE. As shown in Table 4, the recovery rates exceeded 50%, with an overall average of approximately 70%. In this experiment, the recovery rate was higher for subject 3 than for subject 5. This can be attributed to a matrix effect (Table 5). In both subjects 3 and 5, the recovery rate at low concentrations of serotonin-d_4_ was higher than that at high serotonin-d_4_ concentrations. The reason of this result is probably due to the fact that the amount of serotonin (serotonin-d_4_ and endogenous serotonin) contained in the high-concentration samples exceeded the capacity of the SPE columns.

**Table 4:** Table 4. Recovery rates and intraday and interday variance.

Subject 3						
Concentration of Serotonin-d_4_ (μM)	Intraday (*n*=6)			Interday (*n*=5)		
	Recovery rates of SPE (%)	CV of recovery rates (%)	CV of Serotonin/Serotonin-d_4_ area ratio (%)	Recovery rates of SPE (%)	CV of recovery rates (%)	CV of Serotonin/Serotonin-d_4_ area ratio (%)
0.01	81.0±3.0	3.7	4.1	78.1±8.6	11.1	3.9
0.1	79.5±5.2	6.5	4.5	77.3±10.9	14.1	2.5
0.5	63.6±3.7	5.8	2.2	63.9±9.1	14.3	7.5
1	67.4±4.7	6.9	8.8	68.8±11.6	16.9	5.6
Subject 5						
Concentration of Serotonin-d_4_ (μM)	Intraday (*n*=6)			Interday (*n*=5)		
	Recovery rates of SPE (%)	CV of recovery rates (%)	CV of Serotonin/Serotonin-d_4_ area ratio (%)	Recovery rates of SPE (%)	CV of recovery rates (%)	CV of Serotonin/Serotonin-d_4_ area ratio (%)
0.01	65.9±4.8	7.3	4.7	67.7±6.9	10.3	2.5
0.1	58.2±2.5	4.3	2.2	60.8±9.1	15.0	2.5
0.5	58.5±5.1	8.6	3.8	69.0±13.7	19.9	3.9
1	55.9±3.1	5.5	2.3	56.5±10.7	18.8	2.6

**Table 5:** Table 5. Matrix factor and serotonin contents per dry feces of volunteers of Group 1.

Subject	1st		2nd	
	Matrix factor (%)	Content per dry weight (ng/mg)	Matrix factor (%)	Content per dry weight (ng/mg)
1	61.9	1.76	56.3	1.39
2	53.6	1.98	53.1	1.59
3	71.1	0.45	68.7	0.58
4*	64.1	1.81		
5	40.6	1.72		
6	55.0	1.70	60.6	0.30
7	51.7	1.53	57.1	0.65
8	54.2	2.20		
9*	54.6	0.84		
10	55.8	0.64	68.9	1.03
11	57.4	1.61	45.1	1.71
12	55.3	3.77	48.5	2.03
13			64.2	0.81
14			58.5	0.54
15			61.6	1.04
16			60.8	1.03
17			57.8	1.24
18			51.6	1.18
19			57.5	5.53
20			26.8	9.93

* Female subjects

**Table 5b:** 

	Matrix factor (%)	Content per dry weight (ng/mg)
Average±SD	56.2±8.7	1.81±1.88
CV	15.6	—
Range	—	0.30–9.93

Regarding variance, the intraday CV was less than 10%, and the reproducibility was acceptable (Table 4). The interday CV (once every five consecutive business days) was less than 20%, being slightly higher than the intraday CV. This is probably due to the fact that the powdered feces stored in the freezer developed some residual inhomogeneity. Poorly crushed food waste and other materials remained in the powdered feces after homogenization. The fecal samples collected from the stock container at one time for intraday CV evaluation were more homogenous than those collected from the stock container on different days for interday evaluation.

Nevertheless, the CVs of the signal intensity ratio (analyte peak area ratio) of endogenous serotonin and serotonin-d_4_, which was used for the quantification of the endogenous serotonin, were mostly less than 5% (Table 4). Therefore, the accurate quantitative values for the endogenous serotonin concentrations in human feces can be obtained by appropriate correction with serotonin-d_4_ as an IS substance.

### Fecal matrix factors and serotonin quantitative values of Group 1

Table 5 shows the quantitative values for fecal serotonin and MF for 28 fecal samples from 20 subjects. The analyzed samples were prepared from a 200 μL aliquot of the centrifuged supernatant of the fecal solutions (dissolving 20 mg of the powdered feces in 400 μL of the eluting solution) containing 10 mg of powdered feces and finally dissolved in 200 μL of water, so the serotonin content per unit weight of dried feces was determined. As shown in Table 5, the averages of 28 samples were 56.2±8.7% for MF and 1.81±1.88 ng/mg for the content per dry fecal weight. Serotonin levels varied widely among individuals and the CV of MF was 15.6%. In addition, a pair of samples obtained from the repeated collection from eight subjects differed considerably from each other in both serotonin levels and MF values. The maximum difference in MF values for the same individual (subject 10) was 13%. The significant variation in the fecal matrix effect among individuals as well as among samples collected from a single individual makes the stable isotope-labeled IS such as serotonin-d_4_ necessary for quantitation of serotonin in feces.

### Quantitative serotonin values for Group 2

**Figure 2:**
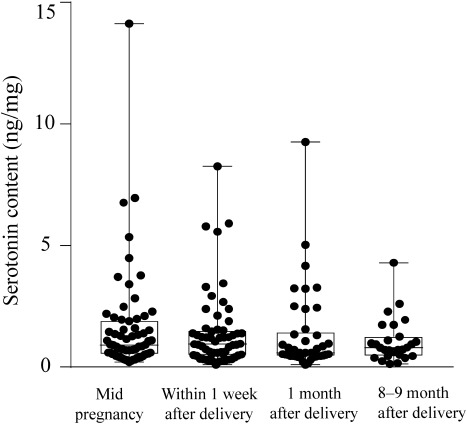
Fig. 2. Serotonin content of pregnant and post-delivery women.

In Table 6, the serotonin content in 192 fecal samples from 76 subjects in Group 2, which was comprised of pregnant and post-delivery women, is presented as well as that in 26 samples from 18 male subjects in Group 1. Figure 2 shows a box-and-whisker plot of the total serotonin contents in feces. On average, the fecal serotonin content in these women decreased with the time from pregnancy to 8–9 months after delivery, although the change was not statistically significant. The tendency for changes in the serotonin contents of 41 subjects whose feces were collected 3 times or more (mid pregnancy, within 1 week after delivery, 1 and/or 8–9 months after delivery) were as follows: In 15 subjects, the serotonin content of the feces was highest at mid pregnancy. In the other 12 subjects, it was highest within 1 week after delivery. In the remaining 14 subjects, it was highest 1 or 8–9 months after delivery. In addition, the mean value for these women was low compared with that of male subjects in Group 1. These issues might be worth future investigation.

**Table 6:** Table 6. Serotonin contents per dry feces of pregnant and post-delivery women of Group 2.

		Serotonin content per dry weight (ng/mg)				
		All	Mid pregnancy	Within 1 week after delivery	1 month after delivery	8–9 months after delivery
Pregnant and post-delivery women	Average±SD	1.42±1.74	1.61±2.12	1.43±1.56	1.35±1.70	1.06±0.89
	Range	0.09–14.13 (*n*=192)	0.20–14.13 (*n*=65)	0.22–5.90 (*n*=59)	0.09–9.27 (*n*=41)	0.13–4.29 (*n*=27)
Males of Group 1	Average±SD	1.84±1.94				
	Range	0.30–9.93 (*n*=26)				

In conclusion, we report on the development of a method for quantifying serotonin levels in human feces using solid phase extraction and column-switching LC-MS/MS. The fecal serotonin content varied substantially among individuals and/or the timing of collection. Therefore, to clarify the physiological and mental significance of fecal serotonin levels, it is necessary to determine the levels in various conditions, and to compare them with the blood serotonin levels. For using fecal serotonin levels as an indicator of the intestinal environment and related health conditions, it will be necessary to further elucidate the factors that affect serotonin levels by comparing the content of fecal serotonin with other indicators of mood, dietary habits and health status.

## Notes

Mass Spectrom (Tokyo) 2020; 9(1): A0081
